# “What is the problem with vaccines?” A typology of religious vaccine skepticism

**DOI:** 10.1016/j.jvacx.2023.100349

**Published:** 2023-07-07

**Authors:** Hanne Amanda Trangerud

**Affiliations:** Department of Culture Studies and Oriental Languages, Faculty of Humanities, University of Oslo, P.O. Box 1010 Blindern, 0315 Oslo, Norway

**Keywords:** Vaccination, Religion, Vaccine hesitancy, Religious vaccine skepticism, Health behavior, Typology

## Abstract

Research has identified religion as one of numerous factors that may contribute to delay or refusal of vaccination. The influence of religion on vaccine decisions may be accidental, or it may involve explicit religious objections. By presenting a typology of religious vaccine skepticism, this article seeks to give a comprehensive overview of the essence of these objections and to clarify differences and similarities between them. This knowledge is useful for policy-makers and others who wish to better understand the influence of religion on vaccine decision-making. The typology consists of five main types: (1) a worldview clash type, in which vaccines do not make sense as a health intervention; (2) a divine will type, which represents a form of passive fatalism; (3) an immorality type, which considers some vaccines unethical because of their production or effect; (4) an impurity type, pointing to ingredients that will defile the body; and (5) a conspiracy type, in which a vaccine plot is targeting a religious group.

## 1 Introduction

Several theories and models have been developed to help explain vaccine decision-making. In 2012, a Working Group on Vaccine Hesitancy was established by the Strategic Advisory Group of Experts (SAGE), the World Health Organization’s leading advisory group on immunization. One of its tasks was to “define vaccine hesitancy and its scope,” [1] and the resulting definition has been an important contribution to a more consistent approach and understanding of the phenomenon:

Vaccine hesitancy refers to delay in acceptance or refusal of vaccines despite availability of vaccine services. Vaccine hesitancy is complex and context specific, varying across time, place and vaccines. It is influenced by factors such as complacency, convenience and confidence [1].

The SAGE definition distinguished 'vaccine hesitancy' from low immunization coverage due to supply and delivery issues. It further pointed to vaccine behavior (delay or refusal) while acknowledging the influence of attitudes, beliefs, and other factors. However, the description of ‘vaccine hesitancy’ as behavior has been disputed by researchers who highlight the psychological nature of hesitancy and argue in favor of understanding the concept as a state of indecisiveness [2], [3]. Regardless of definition, it is clear that a person’s vaccine decisions are the result of a complex process that needs to be understood in its particular context. Research has identified hundreds of factors associated with undervaccination and unvaccination [4]. This article focuses on the influence of religion. In this context, it applies a similar understanding of ‘vaccine hesitancy’ as the SAGE Working Group, with an additional remark that many people who accept vaccines without delay do so with caution [5]. The behavioral perspective was chosen to underline that some people reject vaccination due to religious beliefs without having doubts about their decision.

Religion is listed among the determinants in the SAGE Working Group's Vaccine Hesitancy Matrix1] and is generally recognized by researchers as a potential factor [6], [7], [8]. Noteworthy, studies have found that religion—like other factors—both may and may not be associated with vaccination status. In a global survey, Larson et al. [9] found that 15·4 % of respondents thought vaccines were incompatible with their religious beliefs. However, the study also showed that there was no direct link between a particular faith type and vaccine response, as can be exemplified by the strong resistance towards polio vaccination by Muslims in Nigeria, Pakistan, and Afghanistan contrasted with the Muslim nation of Saudi Arabia which had the lowest religious objection rate (2·3 %) in the survey. This example illustrates that it is imperative to consider the impact of religion in light of other factors, such as politics (both national and international), history (eg, colonialism), and cultural values (eg, gender roles). The relationship between religion and vaccine hesitancy is, in other words, complex and context specific.

While many studies have examined and acknowledged the role of religion in different contexts, there is yet to be found a comprehensive, analytical overview of what makes immunization religiously problematic. There are, however, some insightful overviews of how the major religions deal doctrinally with vaccination [10], [11], [12], [13]. Most frequently cited, Grabenstein's review [10] of religious teachings identified several concerns, yet showed that none of the major faith traditions explicitly disallowed vaccination (with the exception of Christian Science). While informative, a sole focus on doctrines in this regard may overemphasize the role of canonical texts and certain authority structures, thus representing a Western, Christian bias. Such an approach may overlook that practice in some religions is more important than doctrines. The mere existence of different sects and subgroups further testifies that doctrines are often interpreted differently, and members of the same group may look to different authorities to find guidance for their daily lives (including health choices).

To shed light on how religion influences vaccine decisions in practice, this article offers a complete and coherent typology of religious vaccine skepticism (RVS), based on a review of literature pertaining to relevant religious beliefs. The aim is to capture and describe the essence of the various religious objections and to clarify the relationship between them. This knowledge can be useful for policy-makers and others who wish to better understand the influence of religion on people’s vaccine decisions.

**Fig. 1:**
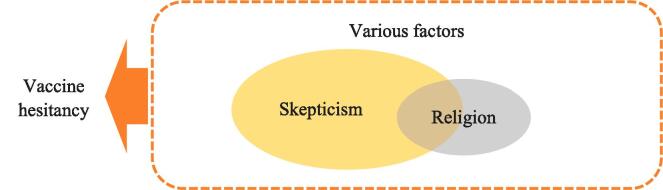
A wide variety of factors may contribute to vaccine hesitancy. Some involve skepticism, which can be either secular or religious in nature (the former pertaining to, for instance, safety concerns). The typology presented in this article deals with skepticism that is somehow related to religion. Other aspects of religion that may contribute to vaccine hesitancy are not included.

RVS here denotes an attitude of doubt or disbelief towards vaccines that originates in, is related to, or is explained as religion. Fig. 1 illustrates how this relates to vaccine hesitancy in general. Note that religion may contribute to delay or rejection of vaccination without involving skepticism. The impact may be accidental, as when a ritual takes place on the day allocated for immunization [14], or indirect, as when a certain lifestyle and limited secular education contribute to an inadequate understanding of the rationale for immunization [15]. In addition, differences between religious groups in vaccine uptake need not result from religious qualities (eg, behavioral regulations) but may be caused by other variations (eg, access to health care). These aspects are not included in the RVS typology, which only covers explicit religious objections.

## 2 Methods

The typology was developed within the academic discipline of the study of religion, based on a literature review of research on religion’s influence on vaccine decisions. The included articles were peer-reviewed, written in English, from different fields (predominantly health sciences and social sciences), and of various kinds (case studies, meta-analyses, systematic reviews, situation analyses). Although religion was not necessarily their primary focus, their findings involved significant religious arguments against vaccination. As the aim of the literature review was to identify as many religious arguments as possible, the search was broad and paid equal attention both to frequently mentioned and to rare objections. For instance, an initial search was conducted in September 2019 in the BioMed Central (BMC) database with the search words “vaccination & religion.” This gave 505 hits (1999–2019), of which about four-fifths did not provide sufficiently detailed information about the role of religion to be relevant (eg, when religion was only mentioned as a demographic variable or simply labeled as a factor). From the relevant articles, all described or cited religious arguments were taken into consideration. Several other articles were identified from the bibliographies, and similar searches were made throughout 2022 in other databases (JSTOR, PubMed, Google Scholar). The main searches were performed with the terms “vaccination/immunization & religion/religious,” and relevant findings were followed up with more specific searches related to, for instance, mentioned groups (eg, “Amish & vaccination”) or beliefs (eg, “vaccines & fatalism”). Since the typology deals with quality (the existence of an objection) and not quantity (the prevalence of the objection), the search was closed when it reached the level of thematic saturation, that is, when further reading revealed no new themes. Finally, relevant review articles [10], [11], [12], [13] were used to control the types to ensure that no significant objection had been overlooked.

The types were structured according to the essence of what is considered problematic with vaccines from the perspective of a religious individual or group, that is, as answers to the question, “What is the problem with vaccines?” The perceived reality of a religious person may involve elements that are not part of the shared human experience, like actors (eg, supernatural beings), acts (eg, rituals), and modes of communication (eg, prayers). The types encompass all these elements but are not specific in the sense that they only involve one particular god, religious act, or similar. The essence captured in each type may hence span across religious traditions and groups.

## 3 Results

The analysis resulted in five main types which are described and exemplified below. The applied examples are intended to illustrate how each type may manifest in practice and to delineate the difference between them but are by no means exhaustive. It should be stressed that the examples only explain how some members of a particular group consider the issue and do not represent the views of the whole group—with the possible exception of some faith healing groups.

### 3.1 Vaccines are irrelevant or destructive (*worldview clash type*)

**Fig. 2:**
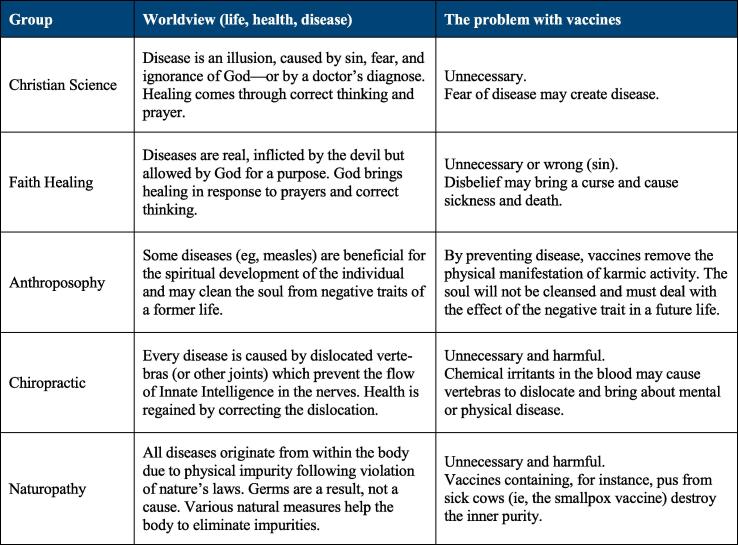
The table exemplifies how some worldviews render immunization irrelevant or destructive. Anthroposophy, which often self-classify as a philosophy, and two samples of what are usually classified as complementary and alternative medicine (chiropractic and naturopathy) are included to illustrate the interplay between gray zone worldviews and vaccine decisions.

The RVS of the first type is based on a worldview in which the causal explanations of life, health, and disease make vaccination inappropriate as a health intervention. Given certain premises, vaccination becomes irrational since it will either be unnecessary or bring about negative consequences. Fig. 2 briefly summarizes the worldview of some groups and shows why vaccines are perceived as problematic.

For instance, members of Christian Science may not only reject vaccines but also other medical interventions due to a belief that diseases are illusions and that only the realization of this can bring about healing. According to this worldview, human beings are the image of God (who is spiritual) and therefore cannot be sick (which is a material phenomenon). However, sin, ignorance of God, and fear—including fear of disease induced by a doctor's visit—may cause disease, and the only remedy is prayer and a correct mindset, that is, convincing oneself that the disease is a delusion [16], [17].

Various Christian faith healing groups reject vaccines for similar reasons. In contrast to Christian Science, these groups acknowledge diseases as real, typically as inflicted by the devil. They nevertheless rely on faith and prayer for healing, only accompanied by rituals, such as anointing with oil or laying on of hands [16]. While doctrinal details vary between the groups, the Faith Assembly, founded by Hobart Freeman in 1963, may serve to illustrate how a faith healing worldview affects the members' health choices. According to Freeman, Satan rules the world through pain, sickness, and medicine, and demons dwell in health institutions, the personnel being satanic priests. Church members are therefore barred from seeking medical treatment, buying health insurance, and using medical devices, like glasses. Healing comes through prayer and by convincing oneself that doubt and pain do not exist, whereas unbelief may hinder recovery [18]. Consequently, parents who believe in faith healing may choose not to vaccinate their children (even in the midst of an epidemic) or seek medical assistance because of fear that their lack of faith may actually harm the child.

While vaccination makes sense from a Western biomedical viewpoint, it does not within these alternative worldviews. This also applies to some semi-religious worldviews, which can fruitfully be treated as belonging to this type. A good example is found in the early tenets of chiropractic, which are still adhered to by about one-fifth of chiropractors [19], [20]. On the one hand, vaccines are considered superfluous since most diseases are believed to be caused by misaligned vertebras pressing on the spinal nerve roots, thus impeding the Innate Intelligence (a vital force necessary for good health) [21]. On the other hand, vaccines are deemed harmful because chemical irritants in the blood can affect the nerves and cause the vertebras to dislocate [22]. There is, in other words, no room for microbes or immune reactions since health is restored and maintained by correcting misaligned vertebras.

The worldview clash type also includes various non-Western worldviews. The perception of polio by the Hausa communities in northern Nigeria may serve as an example. Known as *cutar shan-Inna* (“disease caused by the drinking of Inna”), polio is believed to be a result of a powerful spirit (*Inna*) drinking the blood of the victim’s limb [23]. Healing may come from offering the spirit whatever she informs the traditional healer that she wants in return for the limb (eg, food), accompanied by prayer, incense, and herbal massage. If healing does not occur, it means that the spirit was not satisfied. Within this scenario, the oral polio vaccine—”a few drops of liquid in a [healthy] child's mouth”—makes very little sense [24].

### 3.2 Vaccines interfere with God's will or reveal distrust (*divine will type*)

As with the first type, the RVS of the second type is rooted in the premises of a particular worldview. However, this type differs from the previous when it comes to the role of human beings. In the worldview clash type, people are active. Their chosen course of action is believed to prevent or induce a particular outcome: healing, for instance, may depend on a certain mental process (like convincing oneself that doubt and pain do not exist) and/or a certain activity (such as saying a prayer or correcting the position of the vertebras). In contrast, the divine will type describes the passive acceptance of an outcome that is believed to be decided by God, be it health or disease, life or death. Since God determines the result, it is considered useless or even sinful to try to prevent it.

This type is duly illustrated by orthodox Protestants in the Netherlands, who often base their decision to accept or reject vaccines on religious arguments [25]. Members generally believe in God's foreseeing and guidance over human life [26], and vaccines may be rejected for the same reason: one neither shall nor can interfere with the divine providence. This belief is typically accompanied by a trusting relationship with God and confidence that he will also give strength to endure the diseases he sends [25].

The divine will type is three-pronged, involving belief in (1) God’s protection from disease, (2) God’s sending of disease, and (3) God’s help during disease. Here, trust is clearly key, although to some people, fear might be the other side of the coin. This can be fear of displaying distrust, as some conservative Amish parents explained their choice not to immunize any of their children: “giving shots means I’m not putting faith in God to take care of my children.” [27]. It can also be fear of making a bad decision. Parents who believe that diseases are God’s will but adverse events their own fault may find non-vaccination—that is, doing nothing and leaving the outcome to God—a safer alternative [28], [29].

The divine will type thus represents a form of passive or classic fatalism, the belief that something will occur regardless of one’s intention or behavior [30]. People who hold such views are generally less compliant with expert advice, for instance, about medical treatments and preventive measures [31]. This can be contrasted with active fatalism, which involves both acceptance and active attempts to influence the outcome [30]. This distinction is important since it underscores that fatalistic beliefs do not always lead to vaccine hesitancy. To illustrate, most Muslims believe diseases occur by God’s will, yet studies indicate that this usually does not prevent their seeking medical treatment or prevention [32]. While some reject vaccination on classic fatalistic grounds [33], [34], [35], Muslims often accept vaccines as part of their duty to protect their health [36], [37].

### 3.3 Some vaccines are unethical (*immorality type*)

**Fig. 3:**
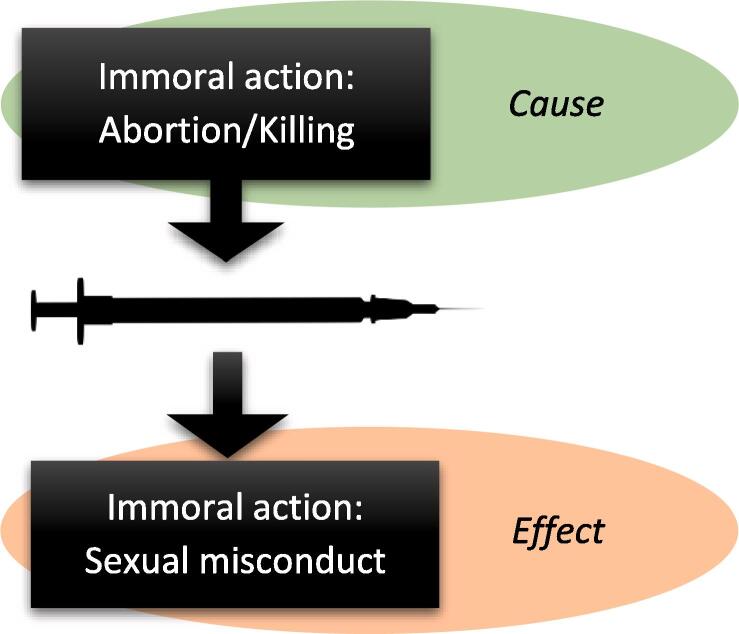
The RVS of the immorality type is either cause-related (involving the production of the vaccine) or effect-related (involving conduct assumed to result from using the vaccine).

The RVS of the third type is related to issues that are considered ethically problematic. Noteworthy, this type does not involve skepticism towards vaccines in general, only towards certain vaccines. As illustrated by Fig. 3, the value judgment of these vaccines is either referring to the production of the vaccine (*cause-related subtype*) or believed to result from the use of the vaccine (*effect-related subtype*).

The cause-related subtype describes skepticism towards vaccines that are deemed unethical because their production somehow is related to the illicit killing or suffering of a being. Here, most attention has been given to cell lines from voluntarily aborted fetuses that are used to grow viruses. Catholics and other Christians who strongly oppose abortion—believing that all human life is sacred—may selectively reject these vaccines [11], [38]. During the COVID-19 pandemic, this objection came into the public eye when it was known that several manufacturers were utilizing fetal cell lines in their attempts to develop vaccines [39].

Due to the belief that cows are sacred, the use of bovine ingredients, like fetal bovine serum, has been identified as a potential concern to Hindus [11]. While underresearched in the context of vaccines, other studies have found reluctance among Hindus to use drugs and medical products that contain bovine material [40], [41]. Historically, reports from 19th century India recount that many Hindus objected to smallpox immunization because of its involvement of cows. Some groups disapproved the pain inflicted on cows by harvesting lymph for the vaccine from the live animals’ skin, whereas lymph from donkeys could be acceptable [42], [43]. During the COVID-19 pandemic, some Hindu leaders requested the Indian president to clarify whether cow blood or similar substances had been used in vaccine production, fearing it could “destroy” their religion [44].

The effect-related subtype involves skepticism towards vaccines that are found ethically unacceptable because they are believed to encourage sexually immoral behavior. This linkage of vaccine and sexual behavior arose since the vaccines in question prevent diseases known to be transmitted through sexual intercourse (human papillomavirus and hepatitis B). Parents may reject these vaccines for their children because they believe they are safe if they follow the principle of abstinence laid down by their religion. Studies have identified this reason for rejecting the HPV vaccine among Christians [45], [46], Jews [47], [48], and Muslims [36], [49]. Closely related, some parents fear that giving these vaccines can in itself trigger early sexual debut or promiscuous behavior [46], [50], [51]. Lastly, the fear of social stigma if one is perceived to need such vaccines may cause hesitancy in cultures where sexual abstinence before marriage is the norm [36], [52].

### 3.4 Vaccines defile the human body (*impurity type*)

The RVS of the fourth type is based on the conviction that some vaccine ingredients are religiously impure—by nature or by preparation—and will defile the human body. This must be distinguished from the immorality type, where, for instance, the use of bovine ingredients is unethical because it is considered wrong to kill or inflict pain on cows. Hindus will therefore not eat cow meat but have no problems with utilizing the cows’ products (eg, milk, urine, or dung) for food, medical treatment, and other purposes [53]. In contrast, the impurity type rejects vaccines because of ingredients that, according to a divine order, are defined as unclean or harmful in essence and therefore will destroy the bodily purity and sanctity that a person is religiously bound to preserve.

Pigs, for instance, are regarded as unclean by Muslims, Jews, and some Christians, such as Seventh Day-Adventists. Several studies have pointed out that to many Muslims, porcine ingredients, like gelatin and trypsin, are major concerns and barriers to immunization which can only be solved by *halal* certification, or proof that the vaccine does not contain prohibited ingredients [54], [55], [56]. While some Islamic leaders and medical experts have declared gelatin to be permissible since it has become clean through a process of transformation [57], not all scholars of Islamic law accept this argument. Porcine ingredients have continued to be controversial to many Muslims, as can be illustrated by the drop in vaccination coverage when Indonesia introduced a new measles-rubella vaccine in 2017. While initially successful, the catchup campaign faced a setback after the Indonesian Ulama Council in Jakarta said the vaccine was forbidden by Islamic law due to porcine ingredients. Although the Council also recognized the vaccine as a means to protect public health, local clerics and parents became skeptical. As a result, the coverage dropped, with the lowest rate of 8 % in one province [58]. Though less prevalent, concerns about unclean ingredients may be a reason for hesitancy to some Jews [59], [60] and some Seventh-Day Adventists [61].

None of the said religious groups will consume products from unclean animals in general (with the possible exception of medical treatments). In contrast, some Christians, who have no problems with eating different types of meat and using other animal products (like medications), believe the body will be polluted if vaccine ingredients from the same animals—along with various chemicals—are injected into it. Typically, they consider the body as “sacred” or as “a temple” that must not be defiled. This idea is quite common among Christians and is generally associated with avoidance of unhealthy behaviors, like drinking and smoking [62]. To some, it is also a rationale for avoiding immunization [10], [63].

### 3.5 Vaccines are used to harm us (*conspiracy type*)

The final type involves RVS that is rooted in conspiracy beliefs, that is, beliefs that vaccines are somehow part of a plot with extensive, negative consequences. Such beliefs need not be related to religion, but as an RVS type, this pertains to vaccine conspiracies that target a religious group or community, as contrasted with other boundary markers, like ethnicity, nationality, or geographical region. There may be some overlap, but a core feature of this type is that religion is used as an identity marker for the alleged targeted group.

Ideas of immunization conspiracies often involve population control through fertility reduction [64], [65], [66] or deadly diseases [67], [68], [69]. In the RVS type, the problem with vaccines is that they function as vectors or camouflage for a secret, harmful substance. Hence, vaccination not only threatens the existence of individuals (due to their religious belonging) but the whole religious community which is targeted. While some people deliberately spread conspiracy rumors to achieve other goals, ordinary people may genuinely believe in them and be struck with fear or anger. The engagement of trusted religious authorities lends credibility to such rumors. Conversely, the environment in which they spread is usually marked by distrust towards authorities outside the group, such as a domestic government, representatives of another religion, a foreign nation, or an international organization. This mistrust often stems from previous negative experiences, which may even have involved vaccines.

The complexity of this type can be illustrated by the boycott of the oral polio vaccine by some of the Muslim-majority states in northern Nigeria in 2003. On the surface, this could resemble a classic religious conspiracy theory with local political and religious leaders warning that the polio vaccine had been deliberately contaminated with anti-fertility agents and HIV viruses as part of a Western plot to reduce Muslim populations worldwide [24]. As has later been pointed out in several in-depth analyses [24], [70], [71], [72], [73], [74], the conspiracy accusations were only a small piece of a larger and much more complex political picture that included years of national struggle for political power between the Christian south and Muslim north; poor health infrastructure and different health priorities in the northern states; and previous negative experiences with Western colonialism, racial prejudices, and a relatively recent vaccine scandal (the Pfizer drug trial of 1996).

The conspiracy type may also include the linking of conspiracy beliefs to a religious narrative. This was, for instance, the case when some Christians rejected the COVID-19 vaccine because they feared it might contain microchips and hence represent “the mark of the beast” [75], [76]. This phrase is part of a biblical passage describing end times events, including persecution of Christians who remain loyal to God in a time of deception and therefore do not “take the mark.” The insertion of the new COVID-19 vaccine into this scenario was enabled by a longstanding popularization, through films and books, of a particular interpretation of this passage, for instance, linking “the mark” to increasingly sophisticated technological innovations, like computer codes. Since receiving “the mark” is detrimental for one’s salvation, Christians may feel they have no choice but to reject the vaccine.

## 4 Discussion and conclusion

While a great number of studies have identified religion as a potential factor that influences people’s vaccine decisions, the content of this factor is often not described [6], [8]. This makes it difficult to tell whether the influence in reality stems from religion or if it is a result of concurrent circumstances, such as the socioeconomic status of the religious group, family, or individual. Even when religion does hinder immunization, this impact may be accidental and not related to skepticism, as when participation in a religious celebration is prioritized over a visit to the clinic. The value of making a clear distinction between RVS and the more random impact of religion on vaccine hesitancy is obvious. For policy-makers and public health workers, it is imperative to know when the solutions to counter vaccine hesitancy are found outside religion—which in the said cases could be the implementation of socioeconomic interventions or simply rescheduling the appointment.

In a similar vein, it is useful to know that RVS is not necessarily reflected in the canonical doctrines of a religious institution. While there are examples of teachings that explicitly prohibit immunization, like those of faith healing denominations, most religious groups do not have straightforward teachings on the matter [10], [11], [12]. However, religious doctrines may still be involved in people’s vaccine decisions, for instance, by defining the permissibility of vaccine ingredients (like pork) or by laying down guidelines for everyday conduct (such as not inflicting pain on cows). Often, one particular doctrine is interpreted differently by different groups, as in the case of the religious duty to preserve life. Consequently, the very same doctrine may be relied on by both acceptors and rejectors of a vaccine. Admittedly, a focus on doctrines may seem rational in contexts where religious exemptions to vaccine mandates are available but only granted when the objection is informed by a religious doctrine (as is the case in some American states). However, reducing RVS to a question of religious doctrines often offers little help in practice.

The presented typology is based on research findings that show how RVS affects vaccine decisions on a practical level. The typology is useful for those who wish to get a more comprehensive understanding of the impact of religion on vaccine hesitancy. The five categories—the worldview clash type, the divine will type, the impurity type, the immorality type, and the conspiracy type—highlight the essence and internal logic of the religious objections and establish the difference between them. This knowledge, in turn, can be helpful for policy-makers and public health workers as different types require different interventions.

For RVS of the worldview clash type, the solution is likely to be found within the worldview in question, that is, by following the rules of logic laid down by the applicable premises. Some of the worldviews mentioned in Fig. 2 provide a loophole for vaccine acceptance themselves: Mary Baker Eddy, the founder of Christian Science, advised parents to have their children vaccinated when it was required by law, informing that they could protect their children from harm by praying and having a correct mindset [77]. Likewise, Rudolf Steiner, the founder of anthroposophy, declared that vaccination would not be harmful if it was followed by spiritual education [78]. Even B. J. Palmer, who succeeded his father in developing chiropractic, claimed that keeping the vertebrae in proper position could protect children from vaccines and other poisons [21]. While such remedies make little sense from a biomedical viewpoint, they may be sufficient for some believers to accept immunization.

The solution to the divine will type, in contrast, could be to aid believers from passive fatalism to active fatalism. This will encourage active participation in caring for one’s health while at the same time respectfully preserving the belief in providence or other external forces that guide destiny. As for the immorality and the impurity types, some believers may be convinced by arguments, such as acceptance in emergency situations with no alternatives available or purification through a process of transformation. Others may not be convinced, and the provision of vaccines with alternative ingredients might be the most convenient solution. In contrast, neither arguments nor alternative vaccines are likely to solve problems arising from the conspiracy type. Here, the building of trust is key—a measure that, in turn, would have little impact on the worldview clash and divine will types. The roots of the conspiracy beliefs must be identified and dealt with individually. In some situations, religious arguments can be helpful, for instance, by reminding believers that “the mark of the beast” according to the biblical text is applied to the hand or the forehead, not to the shoulder.

Bearing in mind that the influence of religion on people’s vaccine decisions is highly complex and context dependent, the exact impact of various interventions are topics for future studies. In general, there is need for more research on RVS, especially when it comes to the prevalence of distinct objections. Further, the extent to which a certain belief results in vaccine rejection should be examined. To different people, the same belief can result in different vaccine decisions, and it can be helpful to discern what other factors influence the result.

## 5 Limitations

The typology was based on available research in English (aided by general knowledge about religions). There is therefore a small possibility that some significant religious views may have been left out. This may not only be related to publication language but also to the possibility that some minor groups have gone under the radar in this context (eg, if their objections are invisible due to low availability of vaccines). The typology nevertheless captures the most important religious objections with the greatest impact globally. Moreover, new categories can easily be added should future research uncover objections that do not fit within any of the existing types.

It can also be discussed whether a non-systematic review was the best way to proceed in order to create a typology of this kind. However, a systematic review would have been less suitable to meet the aim of this project, which was not to provide an overview of the literature but of religious arguments against vaccination. The search therefore had to be broad without predefined categories.

## Declaration of Competing Interest

The authors declare that they have no known competing financial interests or personal relationships that could have appeared to influence the work reported in this paper.

## Data Availability

No data was used for the research described in the article.
